# Retraction: Ginkgo biloba extract EGb 761–induced upregulation of LincRNA-p21 inhibits colorectal cancer metastasis by associating with EZH2

**DOI:** 10.18632/oncotarget.28525

**Published:** 2025-05-08

**Authors:** Tingting Liu, Junzhong Zhang, Zhongqiu Chai, Gang Wang, Naiqiang Cui, Bing Zhou

**Affiliations:** ^1^Department of Integrated Chinese and Western Medicine Surgery, Tianjin University of Traditional Chinese Medicine, Tianjin, China; ^2^Department of Anorectal Surgery, Tianjin Binhai New Area Traditional Chinese Medicine Hospital, Tianjin, China; ^3^Department of Science and Education, Tianjin Binhai New Area Traditional Chinese Medicine Hospital, Tianjin, China; ^4^Department of Oncology, Ruijin Hospital of Shanghai Jiaotong University, Shanghai, China

**This article has been retracted:** Oncotarget has completed its investigation of this paper. The investigation found that Figures 1A, 4A, and 7C contain overlapping and duplicated images, all of which present wound healing assay data from different types of treatment. Additionally, in Figure 4D, the third set of images has overlaps despite representing data of matrigel invasion assay in different cell lines. Figures 1B and 7D contain matrigel invasion assay images from earlier published unrelated papers [1–3]. Figure 4E contains an image of fibronectin WB, which duplicates images in Figure 5C of an already retracted article [4] and Figure 6B of [5]. Figure 4E also duplicates the WB β-actin image in Figure 4D of another earlier paper [6]. Figure 5C, presenting data of the western blots, duplicates panels in Figure 4F from [4]. Almost all WB images in Figures 4E, 5C, 6C and 7B were reproduced multiple times in later publications in different journals. Therefore, the Editorial decision has been made to retract this paper. After the journal contacted the authors with concerns regarding image duplication, corresponding author Naiqiang Cui replied that the results could not be reproduced and all authors agreed with the decision to retract the article.

Original article: Oncotarget. 2017; 8:91614–91627. 91614-91627. https://doi.org/10.18632/oncotarget.21345
